# Differential Activation in Amygdala and Plasma Noradrenaline during Colorectal Distention by Administration of Corticotropin-Releasing Hormone between Healthy Individuals and Patients with Irritable Bowel Syndrome

**DOI:** 10.1371/journal.pone.0157347

**Published:** 2016-07-22

**Authors:** Yukari Tanaka, Motoyori Kanazawa, Michiko Kano, Joe Morishita, Toyohiro Hamaguchi, Lukas Van Oudenhove, Huynh Giao Ly, Patrick Dupont, Jan Tack, Takuhiro Yamaguchi, Kazuhiko Yanai, Manabu Tashiro, Shin Fukudo

**Affiliations:** 1 Department of Integrative Genomics, Tohoku Medical Megabank Organization, Tohoku University, Sendai, Japan; 2 Department of Behavioral Medicine, Tohoku University Graduate School of Medicine, Sendai, Japan; 3 Department of Frontier Research Institute for Interdisciplinary Sciences, Tohoku University Graduate School of Medicine, Sendai, Japan; 4 Translational Research Center for Gastrointestinal Disorders (TARGID), Department of Clinical & Experimental Medicine, KU Leuven, Leuven, Belgium; 5 Laboratory for Cognitive Neurology, Department of Neurosciences, KU Leuven, Leuven, Belgium; 6 Departments of Biostatistics, Tohoku University Graduate School of Medicine, Sendai, Japan; 7 Departments of Pharmacology, Tohoku University Graduate School of Medicine, Sendai, Japan; 8 Departments of Cyclotron RI Center, Tohoku University Graduate School of Medicine, Sendai, Japan; University of California, Los Angeles, UNITED STATES

## Abstract

Irritable bowel syndrome (IBS) often comorbids mood and anxiety disorders. Corticotropin-releasing hormone (CRH) is a major mediator of the stress response in the brain-gut axis, but it is not clear how CRH agonists change human brain responses to interoceptive stimuli. We tested the hypothesis that brain activation in response to colorectal distention is enhanced after CRH injection in IBS patients compared to healthy controls. Brain H_2_^15^O- positron emission tomography (PET) was performed in 16 male IBS patients and 16 age-matched male controls during baseline, no distention, mild and intense distention of the colorectum using barostat bag inflation. Either CRH (2 μg/kg) or saline (1:1) was then injected intravenously and the same distention protocol was repeated. Plasma adrenocorticotropic hormone (ACTH), serum cortisol and plasma noradrenaline levels were measured at each stimulation. At baseline, CRH without colorectal distention induced more activation in the right amygdala in IBS patients than in controls. During intense distention after CRH injection, controls showed significantly greater activation than IBS patients in the right amygdala. Plasma ACTH and serum cortisol secretion showed a significant interaction between drug (CRH, saline) and distention. Plasma noradrenaline at baseline significantly increased after CRH injection compared to before injection in IBS. Further, plasma noradrenaline showed a significant group (IBS, controls) by drug by distention interaction. Exogenous CRH differentially sensitizes brain regions of the emotional-arousal circuitry within the visceral pain matrix to colorectal distention and synergetic activation of noradrenergic function in IBS patients and healthy individuals.

## Introduction

Irritable bowel syndrome (IBS) is a functional gastrointestinal disorder characterized by chronic abdominal pain or discomfort and changes in bowel habits [1]. Psychological disturbance is associated with IBS that is often comorbid with anxiety disorder, panic disorder and mood disorders [2, 3]. IBS patients with phobic anxiety showed more influence to the words with emotional content caused the frontal brain activation and visceral hypersensitivity [4]. Physical or psychological stress aggravates IBS symptoms [5] and evokes colonic motility responses [6]. Stress activates emotional-arousal circuitry in the brain, leading to the production and release of corticotropin-releasing hormone (CRH), a 41-amino-acid peptide, from the paraventricular nucleus of the hypothalamus [7–9]. Hypothalamic CRH secretion results in secretion of adrenocorticotropic hormone (ACTH) from the pituitary, which stimulates the adrenal gland to release cortisol. CRH receptors are widely distributed in the gut as well as throughout the central nervous system [10]. In addition to stimulating the hypothalamic-pituitary-adrenal (HPA) axis [9], CRH induces changes in colonic motility and perception [11] in IBS patients via changes in autonomic outflow. Administration of the nonselective CRH antagonist, α-helical CRH, decreases the exaggerated motility of the colon and visceral pain in IBS patients [12, 13], suggesting that CRH is an important regulator of stress-related brain-gut interactions in IBS.

Functional brain imaging studies with positron emission tomography (PET) and functional magnetic resonance imaging (fMRI) during visceral stimulation have been performed in healthy controls and IBS patients [14, 15]. Brain areas involved in visceral perception and emotion including the insula, cingulate cortex, prefrontal cortices, amygdala, and hippocampus are activated during colorectal stimulation in IBS patients [15–17]. fMRI studies revealed that an orally administered CRH-1 receptor antagonist caused blood-oxygen-level-dependent (BOLD) signal reductions in the amygdala, hippocampus, insula, anterior cingulate and orbital and medial prefrontal cortex of IBS patients and controls during abdominal skin pain expectation [18]. Modulating CRH receptors may alter visceral sensitivity in humans, especially in IBS patients [19]. However, to date, no CRH receptor agonist imaging study of the human brain has been carried out in IBS.

In this study, we investigated the influence of CRH on HPA-axis and brain responses to visceral stimuli in IBS patients and healthy controls. We tested the following hypothesis: in patients with IBS, exogenous administration of CRH is associated with increased responses in both the “visceral pain matrix”, especially the emotional-arousal network, and the HPA- and adrenal axes compared to matched healthy controls.

## Materials and Methods

### Participants

Sixteen male IBS patients (mean age ± SD, 22.3 ± 1.8 years) and 16 sex- and age-matched healthy controls (22.8 ± 2.5 years) participated in this study. Female gonad cycles influence HPA regulation [20] so to rule out the effect of this, only men were included in our study. Sample size was determined by previous brain imaging studies [14, 21]. All IBS patients were diagnosed according to Rome lll criteria [22]. Subtypes of IBS patients were 13 (81%) with diarrhea (IBS-D), 1 (6%) with constipation (IBS-C), and 2 (13%) mixed (IBS-M). Each participant underwent a medical and psychiatric interview and a physical examination. Controls were free from gastrointestinal symptoms. None of the subjects had organic disease, mental disorder, traumatic history, and all were right-handed. No drug was prescribed to the subjects. No IBS patients involved in complementary medicine. Besides, no subjects took any medication, coffee, smoking, or alcohol for a week before the study. Written informed consent was obtained from all subjects. This study was approved by the Ethics Committee of the Tohoku University Graduate School of Medicine, Japan.

Validated questionnaires including the State-Trait Anxiety Inventory (STAI) [23] and the Self-Rating Depression Scale (SDS) [24] were used before the day of the experiment to assess anxiety and depression. There was no significant difference in state anxiety (IBS 41.8 ± 11.9 vs. controls 36.7 ± 8.7, t (30) = 1.39, *P* = .17, Cohen’s d = .49, 95% CI -2.4 to 12.6), trait anxiety (IBS 44.0 ± 11.7 vs. controls 41.1 ± 8.5, t (30) = .81, *P* = .43, Cohen’s d = .29, 95% CI -4.5 to 10.3), or depression (IBS 37.2 ± 7.8 vs. controls 35.9 ± 7.4, t (30) = .47, *P* = .65, Cohen’s d = .17, 95% CI -4.2 to 6.7) between IBS subjects and controls.

### Experimental protocol

**Fig 1:**
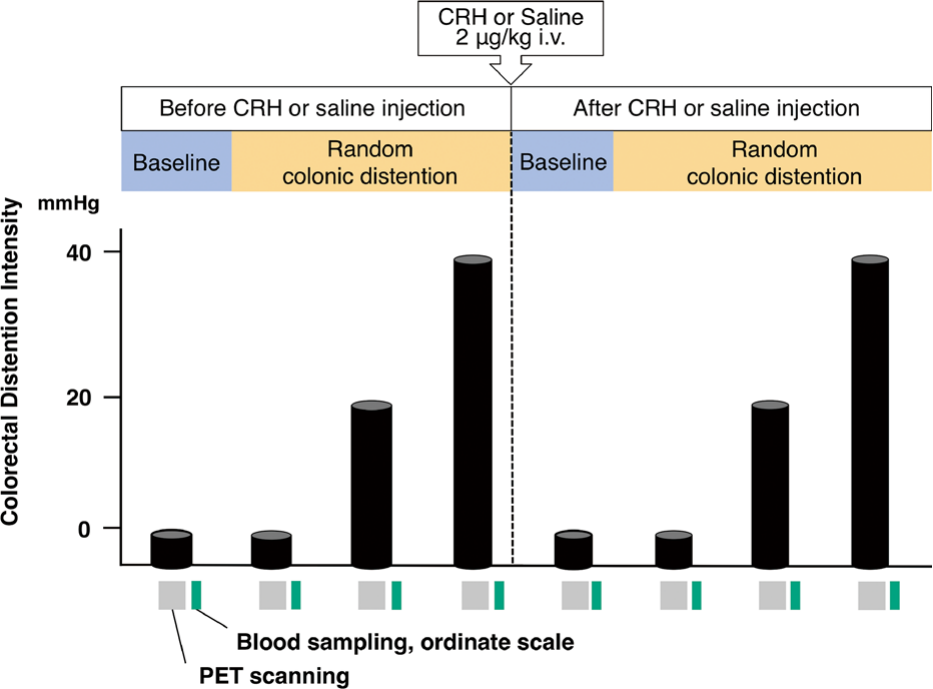
Scanning and distention protocol. A barostat bag was inserted into the colorectum and was inflated with baseline (0 mmHg), no distention (0 mmHg), mild (20mmHg), or intense (40 mmHg) distention for 80s. The time interval between two stimuli was 15 min to allow for radiotracer decay. Conditions were in random order, but baseline was always first. CRH (2μg/kg) or placebo was then injected and same distention protocol was repeated. Radioactive H_2_[^15^O] was injected at bag inflation and then the PET scan was acquired. Blood sampling and ordinate scales for subjective symptoms were measured immediately after each stimulation. Column: baseline, no distention, 20 mmHg or 40 mmHg stimulation.

We used the same barostat protocol as previously described (Supplementary methods) [14, 25]. The intravenous catheter was inserted into each side of cubital vein and then the barostat bag was inserted into the colorectum of each subject 30min before the study. The experiment consisted of two stages: stage 1 without injection of CRH or saline and stage 2 following injection of CRH (Tanabe-Mitsubishi, Osaka, Japan) or saline. Both stages comprised four conditions: baseline (0 mmHg), no distention (0 mmHg), mild (20 mmHg), or intense (40 mmHg) colorectal distention for 80 s each. In each stage, baseline came first, followed by the other three conditions in random order. Subjects were instructed that every condition would be applied in random order. The time interval between two stimuli was 15 min to allow for radiotracer decay. After four scans before injection, half of the subjects in each group (IBS or controls) received either CRH (2 μg/kg) dissolved in saline (2 mL) or saline alone as a bolus, and the same distention protocol was repeated (Fig 1). Subjects were not instructed about the timing of the injection.

The CRH dose used has previously been shown to alter gastrointestinal motility and increase plasma ACTH secretion in rats and in humans [11, 26]. Subjects were blinded to receive either CRH or saline and they were blinded regarding the timing of administration. Blood samples were collected from the cannula in the left cubital vein after each period, and subjective symptoms were evaluated. Plasma and serum were obtained by centrifugation of blood samples at 3,000 rpm for 5 min. These were then frozen and stored at -30°C for later analysis.

### PET scanning and MRI

Each subject underwent one session of structural MR imaging of the brain with a 1.5 T Signa system (General Electric Medical Systems, Milwaukee, WI). A three-dimensional (3D) vascular time-of-flight spoiled gradient echo sequence (50.0/2.4 repetition time /echo time [ms]; flip angle = 45°) was acquired in the axially oriented plane (2 mm thick, 80–90 partitions, 220 mm field of view) and reconstructed into a 256 × 256 × (160–180) matrix (0.859 × 0.859 × 2 mm pixels).

PET acquisition has also been described previously [14, 25]. Briefly, subjects were placed in supine position and were instructed not to move during the session and to keep their eyes closed for the entire scan. rCBF in each subject was measured using a PET scanner in three-dimension sampling mode (HEADTOME V SET-2400W; Shimadzu, Kyoto, Japan). The scanner produced 63 transaxial slices with a thickness of 3.125 mm, an axial field of view of 200 mm, an in-plane resolution of 5.9 mm, full width at half maximum (FWHM), and an axial resolution of 3.9 mm FWHM. For each scan, 30 seconds after receiving injection of approximately 185 MBq of H_2_^15^O intravenously through the right cubital vein, intra-colorectal bag inflation was started. Data acquisition (70 s) began after 10 s of barostat bag inflation.

### Neuroendocrine data

Plasma ACTH and serum cortisol were determined by radioimmunoassay and plasma noradrenaline was measured with high performance liquid chromatography as previously reported [11, 12, 25].

### Ordinate scales for subjective symptoms

Seven subjective symptoms were evaluated on an 11-point ordinate scale from 0 (none) to 10 (maximum)[14]. These were abdominal discomfort, abdominal pain, abdominal bloating, urgency of defecation, anxiety, perceived stress, and sleepiness. Symptoms were checked at the end of each stimulation.

### Statistical analysis

Demographic data was analyzed using SPSS 21.0 (SPSS Inc., Chicago, IL). All data are presented as mean ± SD. Significance level was set at 5%.

#### PET data analysis

Brain imaging data were analyzed using statistical parametric mapping (SPM8, Wellcome Department of Cognitive Neurology, London) within Matlab R2010b (Mathworks Inc., Natic, MA). Preprocessing steps included correction for small movements, warping to Montreal Neurological Institute (MNI) space (using the SPM8 PET template), and smoothing the images with a 3D isotropic Gaussian kernel of 12 mm FWHM. MRI images of each subject were used for coregistration and a brainmask was used to eliminate extracerebral activity. Contributions of each parameter of interest to changes in rCBF were estimated according to the general linear model at voxel level. Average brain activity was fixed arbitrarily at 50 ml/dl/min. ROI analysis was performed in pre-hypothesized regions, using the small volume correction for ROIs in SPM8. Each side (left, right) was considered as a separate ROI. The location and dimension of the ROIs were defined using the Wake Forest University (Winston-Salem, North Carolina) PickAtlas toolbox in SPM8 as follows: amygdala, hippocampus, insula, secondary/primary somatosensory cortex, anterior cingulate cortex, midcingulate cortex, thalamus, posterior cingulate cortex, medial prefrontal cortex, ventrolateral prefrontal cortex, dorsolateral prefrontal cortex, midbrain and pons. Active voxels for each ROI were considered statistically significant at a threshold of Family-Wise Error (FWE) corrected *P* < .05. [27–29]

#### Contrasts

To evaluate effects of CRH on baseline brain activity, the contrast [baseline_after injection_−baseline_before injection_] was subtracted between IBS and controls with CRH injection. To test the CRH effect on brain activity during distention, the contrast [intense distention–baseline]_after injection_—[intense distention–baseline]_before injection_ was compared between CRH and saline injection in each group. To test the hypothesis that IBS subjects showed a different reaction to CRH administration during colorectal distention from controls, the contrast {[intense distention–baseline]_after CRH injection_—[intense distention–baseline]_before CRH injection_}**—**{[intense distention—baseline]_after saline injection_—[intense distention—baseline]_before saline injection_} was compared between controls and the IBS group.

#### Neuroendocrine and behavioral data analysis

The baseline data before injection between IBS patients (n = 16) and controls (n = 16) and after CRH injection between IBS patients (n = 8) and controls (n = 8) were analyzed by Student’s t-test. For baseline comparison before and after CRH injection of IBS patients (n = 8) and controls (n = 8), t-tests were used. GEE [30] was performed using SPSS 21.0 (SPSS Inc., Chicago, IL). The fixed main effects were group (IBS, controls), drug (CRH, saline) and condition (no distention, 20 and 40 mmHg distention) as well as their potential interactions on the dependent variables of interest. Group and drug were between-subject effects and condition was a within-subject effect. A *P* value < .05 was regarded as significant.

## Results

### rCBF Brain imaging

#### Brain responses after CRH/saline injection

**Fig 2:**
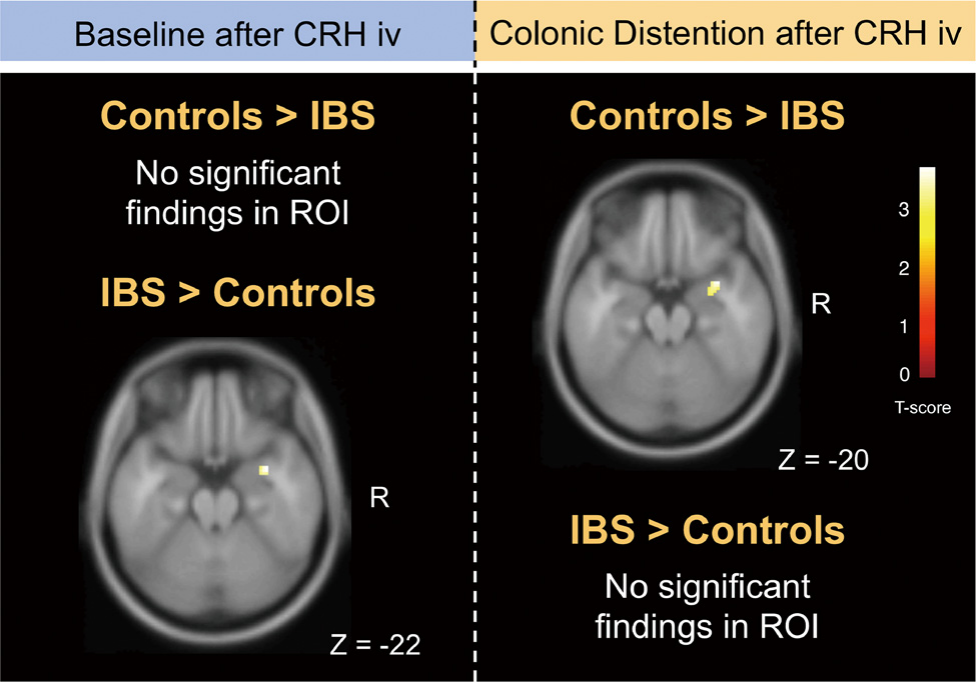
Regions showing significant activation changes. (Left) IBS patients showed significantly more activity than controls in the right amygdala at baseline after CRH injection compared with that at baseline before CRH injection. (Right) Controls showed significantly greater activation than IBS patients in the right amygdala at intense distention after CRH injection compared with saline injection than IBS patients. Results are shown rendered on a single-subject MRI template with axial sections using the threshold *P*_FWE-corrected_ < 0.05 (voxel level, ROI analysis).

We first compared the CRH effect at baseline (i.e. without any distention) between IBS patients and controls. In the comparison between groups we used the contrast baseline after CRH injection–baseline before injection. After CRH injection, IBS patients showed significantly higher activity in the right amygdala compared to controls in a regions of interest (ROI) analysis (t = 3.63, cluster [k] = 42, ROI *P*_FWE-corr_ = .017; local maximum—x: 34, y: 2, z: -22) (Fig 2). No regions were found where the effect of CRH injection was stronger in healthy controls compared to IBS patients. There was no significant difference between IBS patients and controls for the contrast baseline after saline injection–baseline before saline injection.

#### Brain responses to distention after CRH injection compared to saline injection in IBS patients and controls

The healthy controls receiving CRH injection showed significantly stronger activation in the amygdala, hippocampus and middle cingulate cortex at intense distention compared to baseline compared to those receiving saline injection in a ROI analysis (Table 1). No differences in brain responses were found between IBS patients receiving CRH injection compared to patients receiving saline injection (Table 1).

**Table 1:** Effect of CRH on brain activation in response to colonic distentions.

Local max MNI				T score		# Voxels in cluster	ROI *P*_FWE-corr_
Side	x	y	z		Tentative anatomical localization		(voxel level)
**(distention–baseline)**_**after injection**_**—(distention–baseline)**_**before inkection**_							
	**IBS: CRH > Saline**						
					No significant findings in ROI		
	**Controls: CRH > Saline**						
L	-30	-6	-14	3.69	Amygdala	13	.031
R	30	4	-18	3.28	Amygdala	22	.025
L	-30	-8	-16	3.96	Hippocampus	51	.010
L	-10	-18	48	3.80	MCC	7	.031

Increase in rCBF activity in IBS patients and controls during 40 mmHg distention after administration of CRH or saline. MCC: middle cingulate cortex, MNI: Montreal Neurological Institute, L: left, R: right, height threshold: *P*_FWE-corrected_ < .05 [voxel level, region of interest (ROI)].

#### Brain responses to distention after CRH injection compared to saline injection between IBS patients and controls

We then looked for regions differentially activated by distention after CRH injection compared with saline injection between IBS patients and controls. Controls showed a significantly stronger response than IBS patients in the right amygdala during 40 mmHg distention after CRH injection compared with saline injection in ROI analysis (local maximum: x: 34, y: 4, z: -20; t = 3.78, cluster [k] = 34, ROI *P*_FWE-corr_ = .020) (Fig 2). In the same contrast, none of the other ROIs were significantly more activated in IBS patients than in controls.

Brain imaging results before injection of CRH or saline are shown in *SI Results* (S1 File and S1 Fig).

### Neuroendocrine data

**Fig 3:**
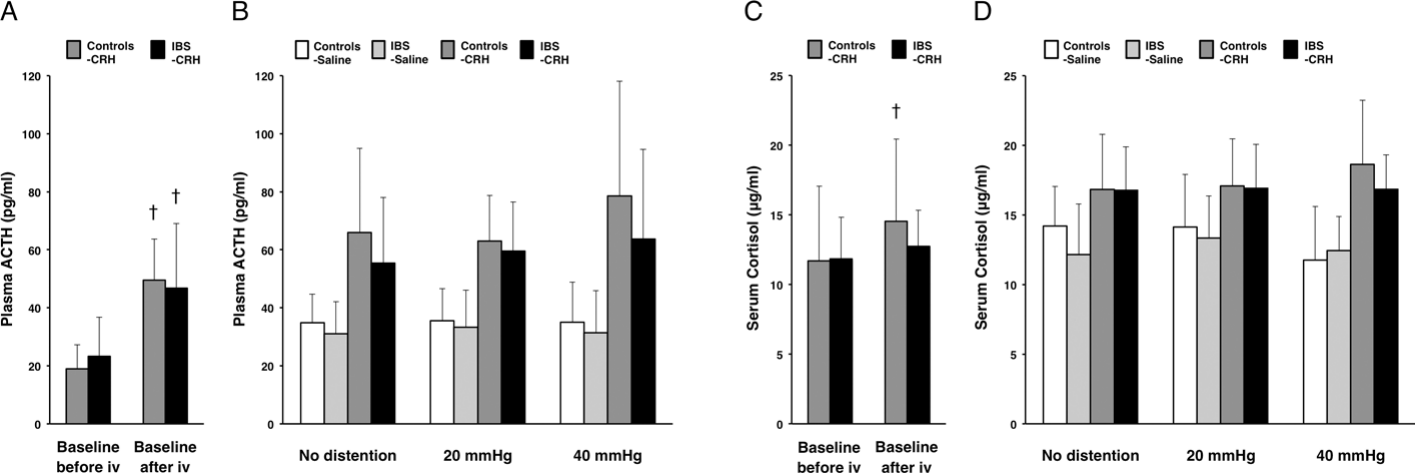
Effects of CRH on the hypothalamic-pituitary-adrenocortical axis. (**A**) Plasma ACTH (pg/ml) at baselines between before and after CRH injection in controls with CRH (n = 8) and IBS patients with CRH (n = 8). iv, intravenous injection. Results represent mean ± SD. ^†^*P* < 0.05, compared with each baseline, paired t-test. (**B**) A significant drug effect and drug × distention interaction in plasma ACTH (pg/ml) during random distention after drug injection was noted between controls with saline (n = 8), IBS patients with saline (n = 8), controls with CRH (n = 8) and IBS patients with CRH (n = 8), analyzed by GEE. (**C**) Serum cortisol (μg/ml) at baselines between before and after CRH injection. ^†^P < 0.05, compared with each baseline. (**D**) Serum cortisol (μg/ml) during random distention after drug injection had a significant drug effect, drug × distention interaction and drug × distention × group interaction by GEE analysis.

Baseline levels of plasma ACTH before injection were not significantly different between IBS patients (27.2 ± 14.7 pg/ml) and controls (31.5 ± 18.2 pg/ml). Baseline levels of serum cortisol before injection were also not significantly different between IBS patients (12.4 ± 3.1 μg/ml) and controls (13.5 ± 5.6 μg/ml). To test the CRH injection effect during baseline, we compared the baseline changes between before and after “drug” (CRH, saline) injection. IBS patients showed significantly higher plasma ACTH levels after CRH injection compared to before injection (*P* = .005) (Fig 3A); this was not the case for serum cortisol levels (*P* = .286) (Fig 3C). Controls showed significantly higher plasma ACTH and serum cortisol levels after CRH injection compared to before injection (*P* = .002 and .048, respectively) (Fig 3A and 3C). After CRH or saline injection, an analysis of neuroendocrine levels during random distention was performed using generalized estimating equations (GEE) [30]. Plasma ACTH showed a significant drug effect (*P* < .001) and drug × distention interaction (*P* = .027) (Fig 3B). However, there was no significant difference between the two groups (IBS, controls). Serum cortisol levels showed a significant drug effect (*P* < .001), drug × distention interaction (*P* < .001) and drug × distention × group interaction (*P* = .001) (Fig 3D).

**Fig 4:**
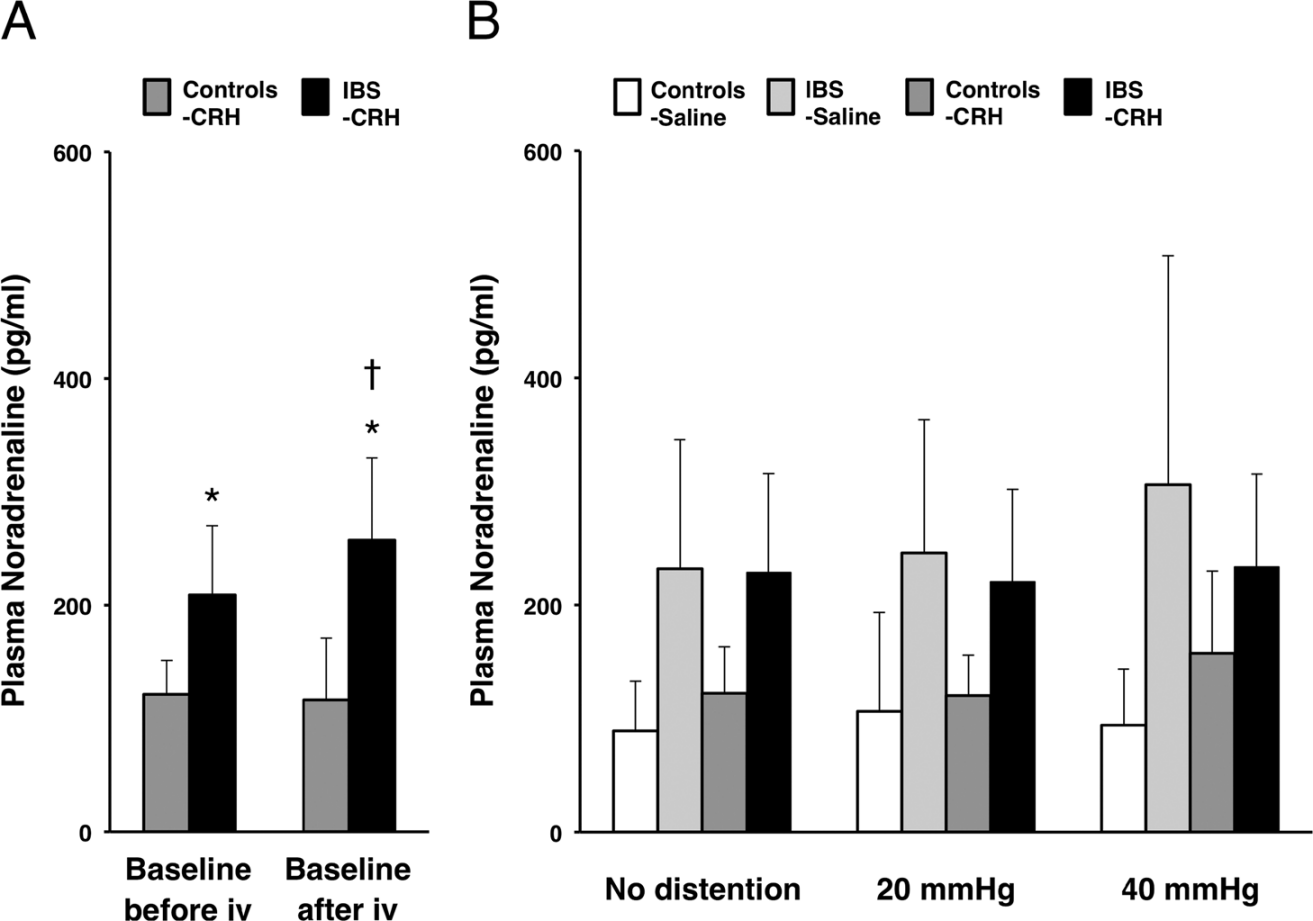
Effects of CRH on the noradrenaline responses. (**A**) Increased basal levels of plasma noradrenaline (pg/ml) were found in IBS patients. IBS patients also showed a significant CRH response compared with basal levels between before and after CRH injection. iv, intravenous injection. Results represent mean ± SD. *P < 0.05, independent samples t-test between controls with CRH (n = 8) and IBS patients with CRH (n = 8). ^†^*P* < 0.05, compared with each baseline, paired t-test. (**B**) Plasma noradrenaline (pg/ml) responses during random distention after CRH or saline injection between controls with saline (n = 8), IBS patients with saline (n = 8), controls with CRH (n = 8) and IBS patients with CRH (n = 8) showed significant group effect, drug × distention interaction and drug × distention × group interaction by GEE analysis.

Baseline levels of plasma noradrenaline before injection were significantly higher in IBS patients (215.3 ± 92.9 pg/ml) than in controls (115.4 ± 39.6 pg/ml) (*P* = .001) and still showed a significant difference between IBS patients and controls after CRH injection (*P* = .001) (Fig 4A). Baseline plasma noradrenaline after CRH injection showed a significant increase from before CRH injection in IBS patients (*P* = .018) but not in controls (Fig 4A). During random distention after drug injection, a significant group effect (*P* < .001), drug × distention interaction (*P* = .037) and drug × distention × group interaction (*P* = .006) were detected (Fig 4B).

### Ordinate scales for subjective symptoms

Comparing baselines between before and after CRH injection, sleepiness score in controls after CRH injection showed a significant decrease from before CRH injection (*P* = .010). During random distention after drug injection, a significant group effect was shown in the abdominal pain scale (*P* < .001), abdominal discomfort (*P* = .029), abdominal bloating (*P* = .003), urgency of defecation (*P* = .019) and sleepiness (*P* = .025) in a GEE analysis. All seven scales showed significant distention effects. A significant drug effect was detected in the sleepiness score (*P* = .025). There was a significant group × distention × drug interaction in urgency of defecation (*P* = .044).

## Discussion

This is the first study demonstrating that exogenous administration of CRH modulates the increases in colorectal distention-induced activation of visceral sensation-related brain regions and neuroendocrine changes in both IBS patients and healthy controls. Moreover, this study demonstrated differential influences of CRH on regional brain activity and neuroendocrine changes during colorectal distention between IBS patients and controls. It is of particular interest to find that CRH increases colorectal distention-induced activity in the amygdala, a key emotional-arousal area within the visceral pain neuromatrix [27, 28, 31, 32] in healthy subjects but not IBS patients. Rather, IBS patients had higher baseline activities in the amygdala after CRH injection than controls.

The central nucleus of the amygdala is involved in fear and anxiety while the lateral part of the amygdala is important for the modulation of nociceptive pain pathways [33, 34], which integrate and send information about sensory input and emotion to the cortex, thalamus, hypothalamus and brainstem [35, 36]. The amygdala contains dense CRH and glucocorticoid receptors and its structure and function in general, and amygdala-related fear responses in particular, are altered by chronic stress [36, 37]. A CRH-1 receptor antagonist dampened effective connectivity of all paths of the emotional-arousal network to and from the amygdala in IBS patients [18]. The findings in our study suggest an intrinsic role of CRH in the amygdala during human visceral nociception and its dysfunction in IBS patients. As the amygdala is more sensitive to exogenous CRH at baseline in IBS patients than controls, this suggests a greater receptor density or an increased receptor affinity [10] in these areas of IBS patients. In IBS patients, colorectal distention likely releases abundant endogenous CRH, which stimulates the amygdala. Our results suggest that the emotional-arousal network has already been activated just after intravenous injection of CRH without visceral stimulation in IBS. This may imply the allostatic load [38, 39] of activation in certain brain regions in IBS due to abnormal CRH receptor distribution.

One of the strengths of this study is the finding of similar dynamics of the plasma noradrenaline and the amygdala responses to administration of CRH in IBS patients and controls. Stress and fear are known to activate subcortical and/or cortical pathways to the amygdala, and these stimuli cause noradrenaline release via the pathway to the locus ceruleus [40]. Although many studies on brain activity during visceral stimulation in humans have been published [14–18], none of them have shown concordant dynamics of the amygdala response and plasma noradrenaline in IBS patients and controls. In this study, IBS patients showed basal plasma noradrenaline levels nearly twice as high as those in controls. Other investigators also reported higher basal levels of noradrenaline in IBS patients compared to healthy subjects [41, 42]. Visceral nociceptive signals spread to the insula, anterior cingulate cortex, and the prefrontal cortices via the lamina I, dorsal column and thalamus [43, 44]. The pathway from the lamina I reaches the amygdala and hypothalamus via the parabrachial nucleus bypassing the thalamus. The amygdalae send excitatory signals to the lateral hypothalamus and eventually activate the sympathetic efferent neurons, which secrete plasma noradrenaline [43]. In a rodent study, CRH injection in the central amygdala induced more noradrenaline release during colonic distention [45]. In this study, IBS patients with CRH injection likely showed a ceiling effect in response to visceral stimulation, both in terms of amygdala activation and plasma noradrenaline release. There are positive feedback loops between CRH neurons in the paraventricular nucleus and locus ceruleus-noradrenaline neurons [40, 46]. However, intracerebroventricular pretreatment with repeated exogenous CRH stimulation attenuates locus ceruleus electrophysiological responsivity [47]; thus, frequent psychological or physiological stress may impair the HPA-adrenergic regulation. The repeated visceral stimulation may gradually change the neuroendocrine and neural networks, finally inducing an allostatic load in IBS patients [38, 39].

In this study, we found that CRH activated the bilateral amygdala at the colorectal distention in controls. Right amygdala in controls then seemed to be more activated than that in IBS patients because amygdala in IBS patients was already activated by exogenous CRH. Again, amygdalae have dense CRH receptors [10,48]. CRH is a peptide of high molecular weight, so it has long been thought to be transported from the brain to the blood but not to penetrate the blood-brain barrier (BBB) [49]. However, under stress, mast cells release mediators that increase vascular permeability near the brain vessels allowing BBB penetration by substances with high molecular weight penetrate the BBB [50]. Thus, peptides and regulatory protein hormones penetrate the BBB by both saturable and nonsaturable mechanisms [51]. The synergistic effect of exogenous CRH with colorectal distention (interoceptive stress) on the amygdala in controls can be explained by considering the BBB as an endocrine interface.

Stress is known to induce CRH release, resulting in pituitary secretion of ACTH and cortisol secretion from the adrenocortex [8, 10]. In this study, CRH administration with colorectal distention increased ACTH and cortisol secretion. Adrenal cortisol secretion is regulated not only by ACTH, but also non-ACTH-mediated mechanisms, such as neurotransmitters, neuropeptides and cytokines [52]. Adrenomedullary components of the sympathetic nervous system are affected by catecholamine or the autonomic nervous system. Repeated stress induces the exaggerated noradrenaline responses which are coupled with HPA activation resulting in cortisol secretion. Glucocorticoid-induced enhancement of memory consolidation requires emotional arousal-induced noradrenergic activation within the amygdala but not the hippocampus [36]. Therefore, emotional arousal-induced noradrenergic activation may be the key source for the HPA dysfunction and its effect to emotional-arousal brain regions, especially the amygdala, may be an important factor to trigger gastrointestinal symptoms in IBS patients.

CRH did not produce changes in symptom ratings except sleepiness and urgency of defecation. We found that abdominal pain changed according to the intensity of colorectal distention and disease (healthy vs. IBS) but did not reflect the CRH effect. Effect of exogenous CRH on regional brain activity may not be sufficiently robust to be reflected in behavioral ratings. As rCBF measurements are physiologically more accurate than those of an ordinate scale or clinical symptoms, our brain imaging results suggest subliminal changes due to CRH administration.

There are some limitations in this study. First, it is possible that study design and patient characteristics may have influenced the results. For example, early life trauma influences HPA-axis responsiveness [53] but this was not relevant to subjects in our study who experienced no traumatic history. Moreover, IBS subjects in this study had no co-morbidity of anxiety or depression. Because IBS patients often have wide range ratio of co-morbidity of anxiety or depression [54], the present findings may be slightly biased in this point. However, functional gastrointestinal disorders including IBS and functional dyspepsia without psychiatric co-morbidity were epidemiologically proven to be as risk factors of anxiety and depressive disorders in the future [55]. We could determine that brain response in IBS patients in this study was not due to co-morbid anxiety or depression but due to IBS pathophysiology per se. Second, low-grade inflammation of the colonic mucosa and elevated cytokines may relate to ACTH hypersecretion [56] but we did not measure plasma cytokines in this study.

In conclusion, healthy individuals showed exaggerated brain activity and pituitary-adrenal responses to the exogenous administration of CRH during colorectal distention. By contrast, IBS patients showed a different reaction in the amygdala and the pituitary-adrenal response from healthy individuals. Our findings suggest a ceiling response in the amygdala during CRH administration and colorectal distention in IBS patients.

## Supporting Information

S1 Fig Supporting Figure 1. Effects of colonic distention on the hypothalamic-pituitary-adrenocortical axis and plasma noradrenaline before CRH administration.
(TIFF)

S1 File Supporting Information Text. This is the supporting methods, results, discussion, references, table, and figure legend.
(DOCX)

## Abbreviations

ACTH: adrenocorticotropic hormone
BOLD signal: blood-oxygen-level-dependent signal
CRH: corticotropin-releasing hormone
FWE: Family-Wise Error
FWHM: full-width at half maximum
GEE: generalized estimating equations
HPA axis: hypothalamic-pituitary-adrenal axis
IBS: irritable bowel syndrome
MNI: Montreal Neurological Institute
MRI: magnetic resonance imaging
PET: positron emission tomography
rCBF: regional cerebral blood flow
ROI: regions of interest
SDS: self-rating depression scale
SPM: statistical parametric mapping
STAI: state-trait anxiety inventory
