# Supplementary material for: Differential Activation in Amygdala and Plasma Noradrenaline during Colorectal Distention by Administration of Corticotropin-Releasing Hormone between Healthy Individuals and Patients with Irritable Bowel Syndrome
Source: PLoS One. 2016 Jul 22;11(7):e0157347. doi: 10.1371/journal.pone.0157347 (PMC4957789; doi:10.1371/journal.pone.0157347)
Supplement: S1 File — This is the supporting methods, results, discussion, references, table, and figure legend. (DOCX) [file pone.0157347.s002.docx]

**Supporting Information Text**

**Methods**

The day before examination, each subject was given a low residue meal and ingested 17 g (13.6%) of magnesium citrate, 75 mg of sodium picosulphate, and 24 mg of sennoside A & B to cleanse the colon that evening (21:00 h). Based on earlier reports [1] and our previous studies [2-4], this bowel preparation was unlikely to affect brain-gut function. Subjects then fasted the next day, during which a polyethylene bag, tightly fixed at both ends to a catheter, was inserted into the colorectum with the distal end of the bag positioned 10 cm from the anal verge and taped in place. The maximal volume of the bag was 700 mL and the maximal diameter and length of the bag at full inflation was 10 cm. The catheter was then connected to the computerized barostat equipment (Synectics Visceral Stimulator, Medtronics Synectics, Shoreview, MN). The barostat bag was inflated on demand at a speed of 38 mL/s. A plastic cannula was inserted into the bilateral median cubital vein, with the right side used for injecting ^15^O-labeled water and the left for sampling blood and infusing saline or CRH. Subjects rested in bed 30 min before the study.

**PET data analysis and Contrasts**

To determine the activation under distention conditions without CRH injection, activity at no distention during random distention was subtracted from that at stimulation with the pressures of 40 mmHg during random distention before CRH or saline injection in each group (16 subjects with IBS and 16 controls) (Supplementary Table 1).

**Results**

**Brain Imaging**

*Brain responses to distention before CRH/saline injection.*

The brain regions significantly activated during 40 mmHg distension compared to baseline in IBS patients and controls are listed in Table S1**.** Between-group comparison during colorectal distension before drug injection showed no significant differences in any of the ROIs.

**Neuroendocrine Data**

GEE analysis of plasma ACTH in before CRH injection during random distention showed a significant distention effect (*P* = .046) (Supplementary Figure 1A). Within group comparison, there was no significant effect in each group. Serum cortisol showed a significant distention effect (*P* = .001) and the comparison within the IBS group only showed a significant distention effect (*P* < .001) (Supplementary Figure 1B). Plasma noradrenaline showed a significant group effect (*P* < .001) and distention effect (*P* < .001) during random distention before drug injection (Supplementary Figure 1C). There was a significant distention effect within the IBS group (*P* < .001) but there was no significant effect within the control group.

**Ordinate Scales for Subjective Symptoms**

During the colonic distention protocol before drug injection, a significant group effect was shown in the abdominal pain scale (*P* < .001), abdominal discomfort (*P* = .010), abdominal bloating (*P* = .001), urgency of defecation (*P* = .004) and perceived stress (*P* = .018) in a GEE analysis. A significant distention effect was detected in the abdominal pain scale (*P* < .001), abdominal discomfort (*P* < .001), abdominal bloating (*P* < .001), urgency of defecation (*P* < .001), perceived stress (*P* < .001) and anxiety (*P* < .001) in GEE analysis. During random distention, a significant group × distention interaction was shown in abdominal pain (*P* = .005).

**Discussion**

We also found that CRH activates the hippocampus at distention in controls. The ventral hippocampus has strong connectivity with both the hypothalamus and amygdala and is associated with learning and memory processes as well as anxiety-related behaviors and stress responses [5, 6]. Previous reports suggest that anxiety-driven hyperalgesia is a primary function of the hippocampus [7-9]. Activation of CRH-1 receptor in the hippocampus relates to conditioned fear and stress-induced learning [10]. CRH plays a crucial role in dendritic spine activity in the hippocampus under stress via CRH-1 receptor which is localized in the spine head [11]. Our study thus suggests that intense stress like colorectal painful distention induces more CRH release and CRH receptor sensitization in the hippocampus as well as amygdala in healthy controls.

Colorectal distention before drug injection in IBS patients slightly induced plasma noradrenaline release and reduced serum cortisol secretion. Two types of cortisol receptors, which are mineral corticoid receptors (MR) and glucocorticoid receptors (GR), are expressed in the brain [12]. During repeated aversive stimulation, MRs and GRs modulate habituation and MR and GR antagonists inhibited the excessive cortisol secretion by CRH challenge test [13]. This may suggest that the MR-mediated cortisol negative feedback system or GRs inhibition of secretion under stress stimuli may alter the cortisol-CRH network.

**Supporting References**

1 Lemann M, Flourie B, Picon L, Coffin B, Jian R, Rambaud JC. Motor activity recorded in the unprepared colon of healthy humans. Gut. 1995;37:649-53.

2 Fukudo S, Suzuki J. Colonic motility, autonomic function, and gastrointestinal hormones under psychological stress on irritable bowel syndrome. Tohoku J Exp Med. 1987;151:373-85.

3 Fukudo S, Nomura T, Hongo M. Impact of corticotropin-releasing hormone on gastrointestinal motility and adrenocorticotropic hormone in normal controls and patients with irritable bowel syndrome. Gut. 1998;42:845-9.

4 Sagami Y, Shimada Y, Tayama J, Nomura T, Satake M, Endo Y, et al. Effect of a corticotropin releasing hormone receptor antagonist on colonic sensory and motor function in patients with irritable bowel syndrome. Gut. 2004;53:958-64.

5 Bannerman DM, Rawlins JN, McHugh SB, Deacon RM, Yee BK, Bast T, et al. Regional dissociations within the hippocampus--memory and anxiety. Neurosci Biobehav Rev. 2004;28:273-83.

6 LeDoux JE. Emotion circuits in the brain. Annu Rev Neurosci. 2000;23:155-84.

7 McKenna JE, Melzack R. Analgesia produced by lidocaine microinjection into the dentate gyrus. Pain. 1992;49:105-12.

8 Ploghaus A, Narain C, Beckmann CF, Clare S, Bantick S, Wise R, et al. Exacerbation of pain by anxiety is associated with activity in a hippocampal network. J Neurosci. 2001;21:9896-903.

9 Tracey I, Mantyh PW. The cerebral signature for pain perception and its modulation. Neuron. 2007;55:377-91.

10 Fukudo S. Hypothalamic-pituitary-adrenal axis in gastrointestinal physiology. In: RJ L, editor. Physiology of the gastronintestinal tract. Oxford: Academic Press; 2012. pp 791-816.

11 Chen Y, Rex CS, Rice CJ, Dube CM, Gall CM, Lynch G, et al. Correlated memory defects and hippocampal dendritic spine loss after acute stress involve corticotropin-releasing hormone signaling. Proc Natl Acad Sci U S A. 2010;107:13123-8.

12 de Kloet ER, Joels M, Holsboer F. Stress and the brain: From adaptation to disease. Nat Rev Neurosci. 2005;6:463-75.

13 Cole MA, Kalman BA, Pace TW, Topczewski F, Lowrey MJ, Spencer RL. Selective blockade of the mineralocorticoid receptor impairs hypothalamic-pituitary-adrenal axis expression of habituation. J Neuroendocrinol. 2000;12:1034-42.

**Supplemetary Tables**

**Supplemetary Table 1.** **Brain activation in response to colonic distention in before CRH or saline injection.**

| Local max MNI | | | | T score |  | # Voxels in cluster | ROI *P*_FWE-corr_ |
| --- | --- | --- | --- | --- | --- | --- | --- |
| Side | x | y | z |  | Tentative anatomical localization |  | (voxel level) |
| **(distention – baseline) _before injection_** | | | | | | | |
|  | **IBS (n = 16)** | | | | | | |
| L | -12 | -34 | 10 | 3.79 | Hippocampus | 22 | .020 |
| L | -28 | 16 | -6 | 4.30 | Insula | 32 | .013 |
| L | -24 | 24 | 10 | 4.06 | Insula | 19 | .019 |
| L | -30 | -28 | 22 | 4.01 | Insula | 17 | .021 |
| L | -40 | 0 | 12 | 3.96 | Insula | 16 | .022 |
| R | 34 | 10 | 6 | 5.17 | Insula | 406 | < .001 |
| L | -14 | -6 | -2 | 5.68 | Thalamus | 250 | .001 |
| R | 4 | -18 | 12 | 4.32 | Thalamus | 170 | .002 |
| R | 2 | -32 | -2 | 5.23 | midbrain | 361 | < .001 |
| R | 18 | -42 | -40 | 4.57 | pons | 24 | .017 |
| L | -4 | -42 | -42 | 4.00 | pons | 24 | .017 |
|  | **Controls (n = 16)** | | | | | | |
| L | -16 | -34 | 10 | 4.55 | Hippocampus | 15 | .024 |
| L | -36 | 2 | 8 | 4.63 | Insula | 177 | .001 |
| R | 34 | 10 | 14 | 5.90 | Insula | 203 | .001 |
| L | -4 | 34 | 2 | 3.61 | ACC | 8 | .030 |
| L | -8 | 6 | 42 | 3.56 | MCC | 6 | .033 |
| L | -14 | -14 | 18 | 6.24 | Thalamus | 858 | < .001 |
| R | 10 | -26 | 8 | 6.53 | Thalamus | 898 | < .001 |
| L | -6 | -14 | -4 | 4.27 | Midbrain | 91 | .003 |
| R | 12 | -24 | -2 | 4.84 | Midbrain | 120 | .002 |
| R | 6 | -40 | -22 | 4.25 | Midbrain | 29 | .014 |
| L | -6 | -40 | -34 | 4.06 | Pons | 110 | .003 |
| R | 20 | -40 | -38 | 4.26 | Pons | 21 | .019 |

Increase in rCBF activity in IBS patients and controls during 40 mmHg distention. ACC: anterior cingulate cortex, MCC: midcingulate cortex, MNI: Montreal Neurological Institute, L: left, R: right, height threshold: *P*_FWE-corrected_ < .05 [voxel level, region of interest (ROI)].

**Figure Legends**

**Supplementary Figure 1.** Effects of colonic distention on the hypothalamic-pituitary-adrenocortical axis and plasma noradrenaline. (**A**) Plasma ACTH (pg/ml), (**B**) serum cortisol (μg/ml) and (**C**) plasma noradrenaline (pg/ml) between before CRH injection in controls (n = 16) and IBS patients (n = 16), analyzed by GEE. Results represent mean ± SD.
